# Development of ErbB2-Targeting Liposomes for Enhancing Drug Delivery to ErbB2-Positive Breast Cancer

**DOI:** 10.3390/pharmaceutics12060585

**Published:** 2020-06-24

**Authors:** Sho Ueno, Min Woo Kim, Gibok Lee, Yong Il Park, Takuro Niidome, Ruda Lee

**Affiliations:** 1 Graduate School of Science and Technology (GSST), Kumamoto University, Kumamoto 860-8555, Japan; 209d5101@st.kumamoto-u.ac.jp; 2 International Research Organization for Advanced Science and Technology (IROAST), Kumamoto University, Kumamoto 860-8555, Japan; nanodream89@gmail.com; 3 School of Chemical Engineering, Chonnam National University, Gwangju 61186, Korea; midcon1@gmail.com (G.L.); ypark@jnu.ac.kr (Y.I.P.); 4 Faculty of Advanced Science and Technology, Kumamoto University, Kumamoto 860-8555, Japan

**Keywords:** targeted therapy, targeting peptide, mTOR inhibitor, immunoliposome, drug delivery system, breast cancer therapy

## Abstract

ErbB2 is a type of receptor tyrosine kinase, which is known to be involved in tumorigenesis, tumor aggressiveness, and clinical outcome. ErbB2-targeting therapy using therapeutic antibodies has been successful in breast cancer treatment. However, the need for repeated treatments and the high cost are major disadvantages with monoclonal antibody therapies. Compared with antibodies, peptides are cheap, relatively stable, and have low immunogenicity. We have developed a highly specific cancer-targeting drug delivery system using a targeting peptide to maximize the therapeutic efficiency of rapamycin and to help prevent drug resistance in ErbB2-positive breast cancer. Physicochemical characterization confirmed the successful construction of ErbB2-targeting liposomes (^ErbB2^Lipo). A comparison of a scrambled peptide (ScrErbB2) with the ErbB2-targeting peptide confirmed that these peptides had similar properties except for the targeting ability. The ^ErbB2^Lipo exhibited higher delivery efficiency in ErbB2 positive BT-474 cells than non-targeting liposomes conjugated with ScrErbB2 (^ScrErbB2^Lipo). This peptide-targeting strategy has the potential to improve the efficacy of chemotherapy in ErbB2-positive cancers.

## 1. Introduction

Cancer is one of the most devastating diseases in the world with numbers of new cases increasing every year. In particular, breast cancer is the most commonly occurring cancer in women. However, the incidence rate varies globally [1]. Different countries have different incidence rates, mortality, and recurrence rates because breast cancer has different genetic variations. ErbB2 is a potent oncogene that plays a critical role in cell proliferation, survival, and growth [2]. ErbB2 is a type of receptor tyrosine kinase normally expressed in epithelial cells, but ErbB2-positive breast cancers are known to express ErbB2 tens of times more than normal cells [3]. Based on a better understanding of its oncogenic action, many studies have shown that the expression level of ErbB2 is directly associated with poor patient prognosis [4].

Targeted therapy is considered an appropriate choice to kill cancer cells more efficiently than non-targeted treatments, and also as a way to overcome systemic toxicity, especially in ErbB2-positive breast cancer patients undergoing conventional systemic chemotherapy, radiotherapy, or invasive surgery [5]. This strategy has been extensively applied by conjugating anti-cancer drugs with various drug delivery systems and has recently been emphasized for precision medicine [6,7,8,9,10]. For example, immunoliposomes conjugated with tumor-targeting molecules have emerged as one approach to maximize the intracellular concentration of drugs in cancer cells using both passive and active targeting [11]. The cellular uptake of the anti-cancer drugs could be considerably improved through receptor-mediated endocytosis when the drugs were delivered to ErbB2-overexpressing cells by the immunoliposomes.

The development of trastuzumab, which is an ErbB2-specific antibody also known as Herceptin, has had a great effect on breast cancer treatment. Many clinical studies targeting ErbB2 have been actively carried out to date and the effectiveness of this approach in ErbB2-positive breast cancer has been demonstrated [12]. However, despite the high affinity and target specificity of therapies based on monoclonal antibodies, the high immunogenicity and cost are often mentioned as disadvantages. To solve these problems, other kinds of targeting molecules, such as aptamers, antigen-binding fragments (Fab) from cleaved antibodies, and peptides, have been investigated [13,14,15]. In the present study, we synthesized a targeting peptide specific for ErbB2. Targeting peptides are cheap and relatively stable than antibodies. A targeting peptide also has lower immunogenicity as well as being easy to conjugate to liposomes because it is a small molecule [16]. However, although targeted therapy via immunoliposomes offers many advantages, there are still many obstacles to be overcome, such as drug resistance. A variety of mutations related to drug resistance may occur in cancer patients. Hence, we need to develop an appropriate strategy to treat the cancer [17].

Rapamycin (Rapa), which is a specific inhibitor of the mammalian target of Rapa (mTOR), is known to possess anti-cancer effects. The PI3K-AKT3-mTOR pathway is a modulator of tumor cell proliferation, progression, and survival, and blocking this pathway is one of the main strategies for treating drug-resistant cancer cells [18,19]. Rapa is an effective drug for cancer therapy. However, several clinical trials have demonstrated that Rapa failed to completely inhibit tumor cells [20,21,22]. In this case, we have developed a highly specific ErbB2-targeting drug delivery system for maximizing the therapeutic efficiency of Rapa in ErbB2-positive breast cancer. First, we synthesized ErbB2-targeting peptides and attached these to a lipid. Second, we developed ErbB2-targeting liposomes to counteract the poor solubility and pharmacokinetics of Rapa. Lastly, the in vitro targetability and therapeutic effects of the ErbB2-targeting liposomes were investigated using ErbB2-positive BT-474 cells. The ErbB2-targeting peptide was effective and this approach has the potential to overcome the limitation of the insolubility of Rapa. In this study, the ErbB2-targeting liposomes directly controlled the PI3K-AKT3-mTOR pathway and may be able to downregulate tumor growth.

## 2. Materials and Methods

### 2.1. Materials

3-[Bis(dimethylamino)methyliumyl]-3H-benzotriazol-1-oxide hexafluorophosphate (HBTU), 1-hydroxybenzotriazole hydrate (HOBt·H_2_O), triisopropylsilane (TIS), α-cyano-4-hydroxycinnamic acid (CHCA), Kaiser test kit (phenol/ethanol, ninhydrin/ethanol, potassium cyanide/pyridine), 1-ethyl-3-(3-dimethylaminopropyl)carbodiimide (EDC), and *N*-hydroxysuccinimide (NHS) were purchased from Tokyo Chemical Industry (TCI, Tokyo, Japan). Dichloromethane (DCM) and *N*,*N*-dimethylformamide (DMF) were purchased from Nacalai Tesque Inc (San Diego, CA, USA). 1,2-Distearoyl-sn-glycero-3-phosphoethanolamine-N-[methoxy(polyethylene glycol)-2000] (DSPE-mPEG2000; DSPE-PEG) and egg L-α-phosphatidylcholine (PC) were purchased from Avanti Polar Lipid Inc. (Alabaster, AL, USA). In addition, cholesteryl hemisuccinate (CHEMS) was purchased from Sigma-Aldrich (St. Louis, MO, USA). Piperidine (PPD) was purchased from Watanabe Chemical Industries (Hiroshima, Japan).

### 2.2. Synthesis of ErbB2 Peptide

A resin having Fmoc-protected phenylalanine (Fmoc-Phenylalanine-Alko Resin) was added to the synthesis PD-10 column (GE Healthcare, Chicago, IL, USA). Then, 2 mL of DCM was added to the column and the apparatus was shaken at 1500 rpm for 1 h for expansion of the resin. After incubation, residual DCM was removed and vortexing was carried out for 1 min with 2 mL of 20% PPD/DMF solution to remove the Fmoc group, which was protecting the *N*-terminus of the amino acid. The reaction solution was collected to check whether the deprotection was successful. Further washing steps were repeated five times with 2 mL of DMF vortexed for 1 min each time. All the elutes were collected in a tube. A spectrophotometer measured the quantification of Fmoc in the collected sample. The absorbance at 300 nm of each sample was measured and the amount of Fmoc group was quantified using the Lambert-Beer equation as ε_301nm_ = 7800 M^−1^cm^−1^. To extend the peptide sequences, 2 mL of DMF mixing solution was prepared with six equivalents of *N*,*N*-Diisopropylethylamine and three equivalents of HBTU and HOBt·H_2_O, the respective amino acids were added to the column, vortexed for 1 min, and shaken at 1500 rpm for 15 min to extend the peptide sequence. A Kaiser test was used to confirm the presence or absence of unreacted amino groups using the ninhydrin reaction. The Kaiser test reagent (10 μL total, phenol/ethanol, ninhydrin/ethanol, potassium cyanide/pyridine) was added to a glass tube. A small amount of resin undergoing elongation of the peptide chain was added to the tube, and the mixture was heated in boiling water for 1 min. Then, the change in color of the resin was observed. A purple color indicates positive and a yellow color indicates negative. At the end of the extension reaction, the synthesized peptides were washed, and dried overnight in a desiccator. A pre-chilled cocktail solution for deprotection at a ratio of trifluoroacetic acid (TFA)/distilled water (D.W.)/TIS = 95:2.5:2.5 was added to the column and stirred for 2 h to cleave the peptides from the resin. To precipitate the peptides, the crude peptides in the tube were immersed in 30 mL of cold diethyl ether, kept in a refrigerator at −20 °C for 10 min, and centrifuged at 15,000 g for 5 min at 4 °C. The supernatant was discarded. This procedure was repeated three times for purification and the peptides were dried overnight in a desiccator.

### 2.3. HPLC and Matrix-Assisted Laser Desorption Ionization-Time-of-Flight (MALDI-TOF) Mass Spectroscopy Analysis

The peptide purity was confirmed by C18 analytical reverse-phase high-performance liquid chromatography (HPLC). The mobile phase was changed as follows: 5–95% acetonitrile (ACN) containing 0.1% TFA vs. deionized water containing 0.1% TFA over 20 min at a flow rate of 1.0 mL/min. HPLC detector wavelengths were set at 220 nm for detection of the peptide bonds.

Matrix-assisted laser desorption ionization-time-of-flight (MALDI-TOF) mass spectroscopy can determine the exact molecular weight of a synthesized peptide. Purified peptide and matrix solution, CHCA, was dropped on a target plate for MALDI-TOF mass spectroscopy and dried in a desiccator. Then, mass spectrometry was performed. The matrix solution was prepared by mixing the solvent with 0.1% TFA aqueous solution and 0.1% ACN solution at a ratio of 1:1 and CHCA was added to the solution until the solution was saturated.

### 2.4. Peptide-Lipid Conjugation and Nuclear Magnetic Resonance (NMR) Analysis

To conjugate the ErbB2 peptide with the lipids, NH_2_-ErbB2-NHS was first synthesized. ErbB2 (11.8 mM), EDC (0.3 mM), and NHS (3 mM) were dissolved in 1.5 mL DMF and stirred for 6 h at room temperature. After the synthesis of NH_2_-ErbB2-NHS, DSPE-PEG-NH_2_, and NH_2_-ErbB2-NHS were mixed together and stirred overnight. The solution was precipitated using diethyl ether and lyophilized. In this condition, the peptides might be conjugated not only with DSPE-PEG-NH_2_ but also coupled inter-molecules because it has two primary amine groups and one carboxyl group in one molecule. However, peptides successfully conjugated with DSPE-PEG-NH_2_ should be anchored in liposomes, and it would act as a ligand. Then, the ErbB2 peptide, DSPE-PEG-NH_2_, and DSPE-PEG-ErbB2 were characterized by proton nuclear magnetic resonance (^1^H NMR) analysis. ^1^H NMR spectra were recorded on a 400 MHz Year Hold Magnet (400JJYH, JEOL Ltd., Tokyo, Japan). All NMR samples were dissolved in DMSO-*d*_6_.

### 2.5. Liposome Preparation and Characterization

Liposomes were prepared by the thin-film hydration method as previously described [23]. Egg PC, CHEMS, and DSPE-PEG in 3 mL of chloroform were mixed at a lipid molar ratio of 8.7:9.2:1. Rapa (5:1, a lipid to drug weight ratio) in 1 mL of methanol was also added to a round-bottom flask. Then, the organic solvent was evaporated by a rotary evaporator at 50 °C under 100 hPa. The remaining solvent was fully removed in the fume hood and the dried lipid film was fully hydrated with 2 mL of water solution containing 0.2 mg/mL of DSPE-PEG-ErbB2 to give a total lipid concentration of 2 mg/mL, which was followed by sonication (Q125, QSonica Llc., Newtown, CT, USA) for dispersion at 50% the amplitude (approximately 60 μm) for 10 min. The crude liposome solution was filtered through a hydrophilic RephiQuik nylon syringe filter with 0.45 µm pore (Rephile Bioscience Ltd., Boston, MA, USA) to remove un-encapsulated drugs and aggregates larger than the filter pore. The particle size, polydispersity index (PDI), and zeta potential were measured using dynamic light scattering (DLS) with a Zetasizer Nano ZS (Malvern Instruments Ltd., Malvern, Worcestershire UK). The stability of the liposome was analyzed for 5 days under phosphate-buffered saline (PBS) (pH 7.4, 6.5, and 5.0) at 37 °C. All measurements were repeated three times. In addition, the morphology of the liposomes was also observed by a transmission electron microscope (TEM), JEOL-2100F (JEOL Ltd.). Specimens on the carbon-coated copper grid were stained following the conventional negative staining procedure with phosphotungstic acid. Rapa-liposome were loaded in 100 kDa Slide-A-Lyzer Mini dialysis tubes (Thermo Fisher Scientific Inc., USA) and were placed at 37 °C in 1 L of PBS solution (pH 7.4 and pH 6.5). At a designated time, the tubes were taken out and centrifugated (*n* = 3 each time point). The samples were analyzed by using a spectrophotometer at 290 nm.

### 2.6. Cell Culture

The human breast cancer BT-474 cell line was purchased from American Type Culture Collection (ATCC, Manassas, VA, USA). BT-474 cells were maintained in Roswell Park Memorial Institute (RPMI) 1640 medium (Wako Pure Chemical Industries, Osaka, Japan) supplemented with 10% fetal bovine serum (Wako Pure Chemical Industries), and 1% penicillin/streptomycin (Wako Pure Chemical Industries) in a humidified atmosphere of 95% air and 5% CO_2_ at 37 °C.

### 2.7. Western Blotting

The expression of the ErbB2 receptor on cells was evaluated by Western blotting. MDA-MB-231 and BT-474 cell protein was extracted using RIPA buffer. The protein concentration was determined using a bicinchoninic acid (BCA) assay, and electrophoresis was performed in 5% polyacrylamide gel (30 μg/lane). The proteins were transferred to nitrocellulose membranes and incubated with mouse monoclonal ErbB2 antibody (185 kDa, 1:1000). The mouse monoclonal glyceraldehyde 3-phosphate dehydrogenase (GAPDH) antibody (37 kDa, 1:2000) was used as an internal control. The signal was visualized using enhanced chemiluminescence (ECL, GE Healthcare) solution and detected by Chemidoc (Fusion Solo, Vilber Lourmat, Collégien, France).

### 2.8. Flow Cytometry

The cellular binding was analyzed by flow cytometry. The BT-474 cells (5 × 10^5^ cells) trypsinized with a trypsin/EDTA solution were treated with rhodamine B-conjugated liposomes in serum-free media for 30 min. After incubation, the suspended cells were washed twice with PBS (pH 7.4) and then fixed with 4% paraformaldehyde (PFA) for 15 min. After washing twice, the binding efficiency of the liposomes to target cells was analyzed using a FACSCalibur flow cytometer (Becton Dickinson, Sumter, SC, USA).

### 2.9. Confocal Microscopy

The cellular uptake was analyzed by confocal microscopy. BT-474 cells and MDA-MB-231 cells (5 × 10^5^ cells) were seeded on 35-mm confocal dishes. After 48 h, the cells were treated with rhodamine B-conjugated liposomes (20 μg/mL) in media for 3 h at 37 °C. After incubation, the cells were washed twice with PBS (pH 7.4), and then fixed with 4% PFA for 15 min. The nuclei were stained with Hoechst 33342 solution (1000:1). The ErbB2-mediated endocytosis was confirmed by blocking the assay. BT-474 cells were seeded in 35 mm^2^ glass-bottom dishes at 5 × 10^3^ cells and incubate for 2 days. An anti-ErbB2 antibody (5 μg) was preincubated for 6 h and liposomes were treated for 3 h. The cells were fixed and counterstained with Hoechst 33342. The cell images were obtained using confocal microscopy (Leica, Wetzlar, Germany) with a 40X oil lens. Hoechst 33342 was excited with a 405-nm laser, and emission using a 455–475-nm band-pass filter. Rhodamine B was excited with a 561-nm laser, and emission using a 600–630-nm band-pass filter. Images were analyzed by Leica software and Fiji.

### 2.10. Cellular Toxicity

The cellular cytotoxicity was analyzed by the MTT assay. The BT-474 and MDA-MB-231 cells were seeded at 1 × 10^4^ cell/well in a 96-well plate and incubated for 24 h. Cells were treated with 10 different concentrations (0.001–225 μM) of ^ErbB2^Lipo or ^ScrErbB2^Lipo for 72 h. MTT absorbance was measured using a spectrophotometer and the IC_50_ values were calculated by Graphpad Prism.

## 3. Results and Discussion

### 3.1. Characterizaation of the ErbB2 Peptide

**Figure 1:**
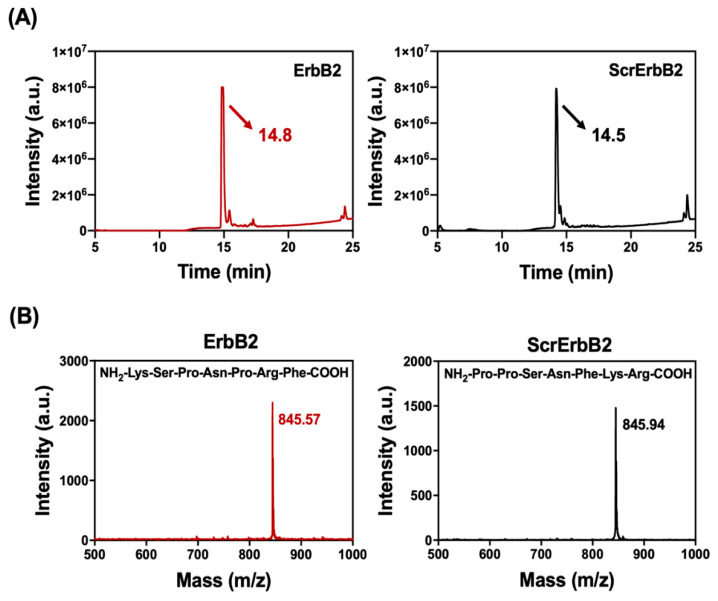
(**A**) The absorbance measured at 220 nm is representative of peptide bonds. A distinct peak confirmed the purity of the peptide synthesized by solid-phase peptide synthesis. The ErbB2 and ScrErbB2 are indicated by arrows. (**B**) Matrix-assisted laser desorption ionization-time-of-flight (MALDI-TOF) mass spectroscopy spectra of purified peptides. Samples were collected at 14–15 min by high-performance liquid chromatography (HPLC). The peaks corresponding to [M + H]^+^ of the peptides ErbB2 (KSPNPRF) and ScrErbB2 (PPSNFKR) had *m*/*z* values of 845.57 and 845.94, respectively (*m*/*z* expected: 844.45).

Targeting peptides bind to their target tumor antigen with high specificity and can be used for targeted drug delivery [24]. The binding affinity of a targeting peptide can be as potent as an antibody and is determined by the sequence of peptide. ScrErbB2 was constructed by rearranging the original peptide sequence and used as an experimental control in all studies. The ErbB2-targeting peptide and ScrErbB2 were synthesized by solid-phase peptide synthesis (SPPS), which is the most commonly used methodology for the synthesis of peptides [25]. The introduction rate was evaluated after each Fmoc-deprotection to estimate the Fmoc level that was eluted during peptide synthesis (Table S1). Because the introduction rate of each amino acid was around 100%, the elongation of the peptide was considered successful. Then, HPLC purified the peptides. Clear signals of the ErbB2 and ScrErbB2 peptide peaks were detected at 14.5 and 14.8 min, respectively (Figure 1A). Both peptides have almost the same retention time, which implies that they are composed of the same amino acids except in a different sequence. Further evaluation of synthesized peptides was conducted by MALDI-TOF mass spectroscopy. When we analyzed the purified sample, molecular ion fragments at *m*/*z* 845.57 for ErbB2 (KSPNPRF) and 845.94 for ScrErbB2 (PPSNFKR) were found, which agreed with the expected values for the synthesized peptides (Figure 1B). These results demonstrated the successful synthesis of the ErbB2 peptide, which was then used in the following experiments.

### 3.2. Characterizaation of Peptide-Lipid Conjugates

**Figure 2:**
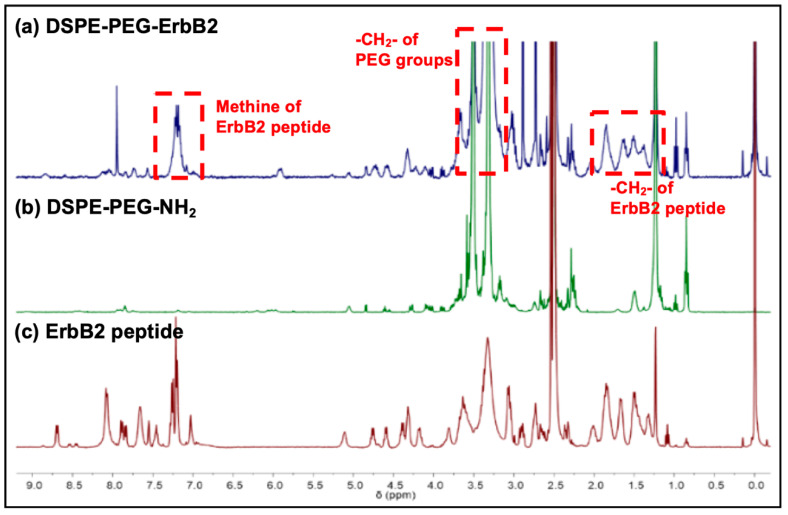
The ^1^H NMR spectra of (**a**) DSPE-PEG-ErbB2, (**b**) DSPE-PEG-NH_2_, and (**c**) the ErbB2 peptide. The red dashed squares indicate the chemical shifts of the methine and methylene protons of the ErbB2 peptide and the methylene protons of DSPE-PEG-NH_2_.

Functional modification of liposomes can be achieved by incorporating polyethylene glycol (PEG)-lipids, which can enable manipulation of the drug encapsulation and a prolonged blood circulation time in the body [26]. A library of PEG derivatives can be used to define the extent of conjugation with tumor-specific ligands on the surface of the liposomes [27]. After the interaction between a specific receptor-expressing tumor cell and its ligand, recognition and internalization into the cytosol can occur via receptor-mediated endocytosis, which is more effective than non-specific uptake [11]. Consequently, this strategy can affect the pharmacokinetics of a particular drug and lead to enhanced therapeutic effects. In the present study, conjugation between DSPE-PEG-NH_2_ and the ErbB2 peptide was affected by a reaction of the peptide with EDC and NHS to form an acyl amino ester, which, subsequently, was reacted with the amine group of the lipid to yield an amide bond (Supplementary Materials Figure S1). The ^1^H NMR spectra of the ErbB2 peptide, DSPE-PEG-NH_2_, and DSPE-PEG-ErbB2 are shown in Figure 2. A broad chemical shift (δ = 1.5–2.0 ppm) of the methylene (–CH_2_–) protons was observed because the methylene protons were adjacent to the amine groups in the ErbB2 peptide. In addition, the methine protons (δ = 7.20 ppm) of the aromatic ring were observed. The spectra of DSPE-PEG-NH_2_ showed the methylene (–CH_2_–) protons (δ = 3.51 ppm) of PEG and the methyl (–CH_3_) protons (δ = 0.85 ppm) of the long alkyl chain. The DSPE-PEG-ErbB2 spectra showed broad chemical shifts (δ = 1.5–2.0 ppm) of the methylene protons from the ErbB2 peptide, and the methylene protons (δ = 3.51 ppm) from the PEG groups were also observed. The spectra of DSPE-PEG-ErbB2 showed the methine protons (δ = 7.20 ppm) of the aromatic ring in the ErbB2 peptide. Thus, these ^1^H NMR spectra demonstrated the successful synthesis of DSPE-PEG-ErbB2.

### 3.3. Characterizaation of ErbB2-Targeting Liposomes

The delivery formulations of hydrophobic drugs are generally restricted to antibody-drug conjugates (ADCs), peptide-drug conjugates (PDCs), micelles, and PEGylated liposomes [28,29,30,31]. Hydrophilic PEG linkers play an important role in all these formulations to improve the solubility of drugs. However, some drugs, such as ADCs and PDCs, might be cleared by renal filtration after drug administration because of their small size [32]. The use of liposomes as drug delivery systems for hydrophobic drugs allows the efficient delivery of a sufficient amount of the anti-cancer agents to the specific tumor site. Considering the smaller size of ADCs and PDCs, approximately 10 nm in diameter, liposomes, which are 100–150 nm in diameter, can change the biodistribution of a drug while circulating for a long time in the blood.

**Figure 3:**
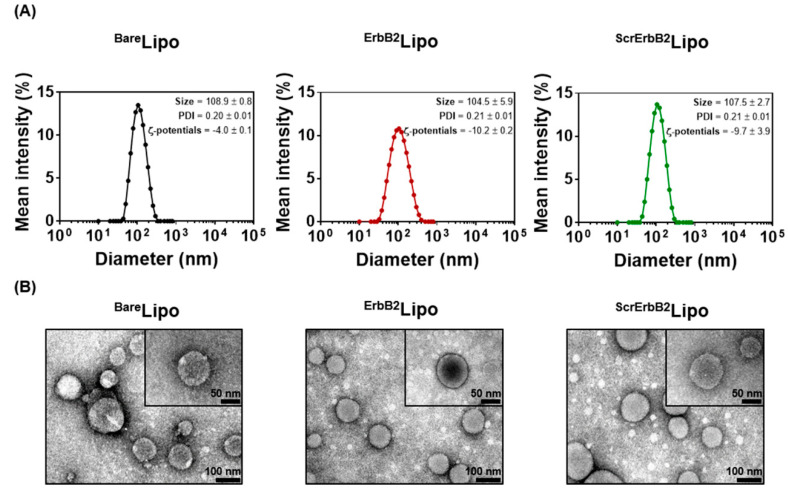
Physicochemical characteristics of the liposomes. (**A**) The particle size, polydispersity index (PDI), and zeta-potential values were measured using dynamic light scattering. (**B**) Transmission electron microscope images of ^Bare^Lipo, ^ErbB2^Lipo, and ^ScrErbB2^Lipo.

The particle size, zeta-potential, PDI, and particle shape of the liposomes were measured immediately after preparation by DLS and transmission electron microscopy (TEM). Liposomes without the targeting peptide ^Bare^Lipo were 108.9 ± 0.8 nm in diameter with a PDI value of 0.20 ± 0.01, while ^ErbB2^Lipo and ^ScrErbB2^Lipo were 104.5 ± 5.9 and 107.5 ± 2.7 nm in diameter with PDI values of 0.21 ± 0.01 and 0.21 ± 0.01, respectively (Figure 3A). The overall particle size and PDI values were almost the same, which indicated that the peptide on the surface of the liposomes did not affect the particle size. The surface charge was slightly decreased in the peptide-conjugated liposome groups, which is believed to be because of the additional PEG-peptide lipids. TEM observations were carried out to confirm the shape of liposomes. The size stability was confirmed up to 5 days in various pH values of PBS. The liposome showed no significant size changes at pH 7.4. However, at pH 5.0, its liposome size increased rapidly from 140 to 220 nm after 5 days of incubation (Figure S2A). Compare to a pH of 5.0, at a pH of 6.5, the size increase was not as sharp. However, it becomes unstable than a pH of 7.4 (Figure S2B). The TEM images revealed that all the liposomes were spherical with a diameter of approximately 100 nm, corresponding to the results of the DLS analysis (Figure 3B). In our previous study, the attachment of ligands, such as antibodies and aptamers, on the surface of liposomes, affected the particle size, which resulted in differences in biodistribution. Each ligand had a different K_d_ value, which indicated possible changes in their binding affinity for the target receptor [23]. The targeting peptides are smaller and have a lower molecular weight (<1 kDa) than monoclonal antibodies (10–15 nm in diameter, approximately 150 kDa) or aptamers (3–5 nm in diameter, approximately 30 kDa) [33]. Previously, the particle size has been observed to increase by approximately 10 nm in diameter after aptamer conjugation and by 15 nm in diameter after antibody conjugation. Although these do not appear to be dramatic increases, recent animal studies have emphasized the dilemma posed by the different enhanced permeability and retention (EPR) effects observed in rodent models [34]. As argued by Danhier, human tumor microenvironments differ dramatically from murine microenvironments. Hence, drug delivery systems with particles larger than 100 nm can perform much differently in clinical trials in humans compared with murine models. In this respect, the use of peptide ligands has a great advantage in maintaining the properties of liposomes, even though peptide ligands cannot yet fully surpass antibodies in terms of affinity.

To develop a treatment for ErbB2-positive breast cancer, Rapa, which is a well-known representative mTOR inhibitor, was encapsulated in three types of liposomes including bare liposomes (^Bare^Lipo), ^ErbB2^Lipo, and ^ScrErbB2^Lipo. Rapa has shown reliable anti-cancer effects and is still being investigated as a potential anti-cancer treatment [35]. For the preparation of liposomes encapsulating rapamycin, the liposomes were synthesized with a drug to a lipid weight ratio of 1:5, and the final encapsulation efficiency and loading efficiency were 68.0% ± 1.7% and 11.9% ± 2.3%, respectively (Figure S2C). The in vitro drug release profiles of Rapa were confirmed under the physiological condition at a pH of 7.4 and about 30% was released up to 72 h. However, at a pH of 6.5, burst release (about 40%) was observed within 24 h and 70% od Rapa released within 72 h (Figure S2D). It suggests that CHEMS-based liposomes can control release under an acidic environment, such as a tumor.

### 3.4. Cancer Cell-Specific Binding of ErbB2-Targeting Liposomes

**Figure 4:**
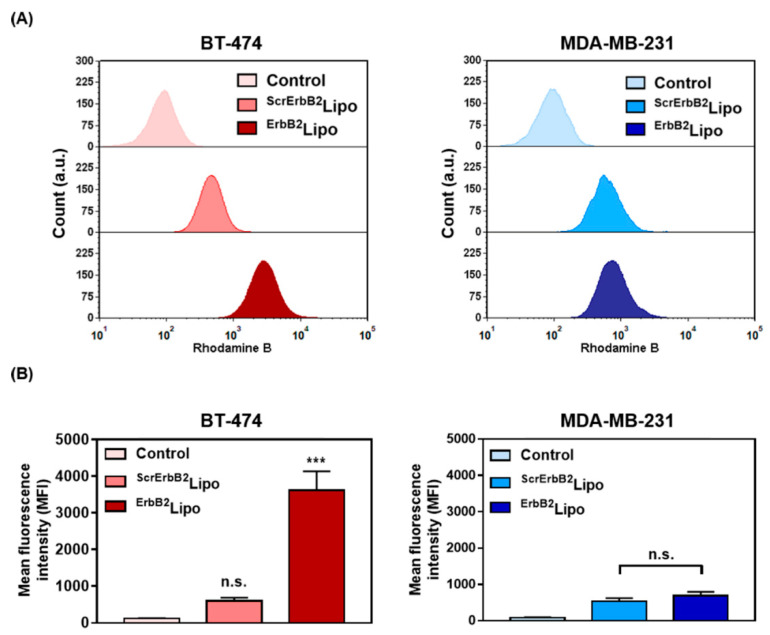
Analysis of binding between rhodamine B-labeled liposomes and breast cancer cells using flow cytometry. BT-474 and MDA-MB-231 cells were treated with control, ^ScrErbB2^Lipo, and ^ErbB2^Lipo. (**A**) Plots of flow cytometry results and (**B**) quantitative analysis of Figure 4A.

To confirm that the liposomes can actively target cancer cells, the binding between the peptide and cancer cells was evaluated. By coupling an appropriate tumor-targeting peptide, the liposomes were predicted to effectively deliver cargos to ErbB2 receptor-positive BT-474 cells, and not to MDA-MB-231 cells, which are known to be ErbB2 receptor-negative cells [36]. Western blot results showed that the ErbB2-receptor expression levels in each cell line were clearly different (Figure S3). The ErbB2-specific binding of ^ErbB2^Lipo and ^ScrErbB2^Lipo containing rhodamine B was examined by flow cytometry (Figure 4). BT-474 cells treated with ^ErbB2^Lipo showed peaks that had a distinct shift to the right compared with control or ^ScrErbB2^Lipo (Figure 4A). The same analysis with MDA-MB-231 cells showed that both ErbB2Lipo and ScrErbB2Lipo were weakly bound to the cells. A comparative analysis of liposome uptake revealed that BT-474 cells treated with ^ErbB2^Lipo showed the highest mean fluorescence intensity value because ^ErbB2^Lipo targeted the ErbB2 receptor-positive cells (Figure 4B).

**Figure 5:**
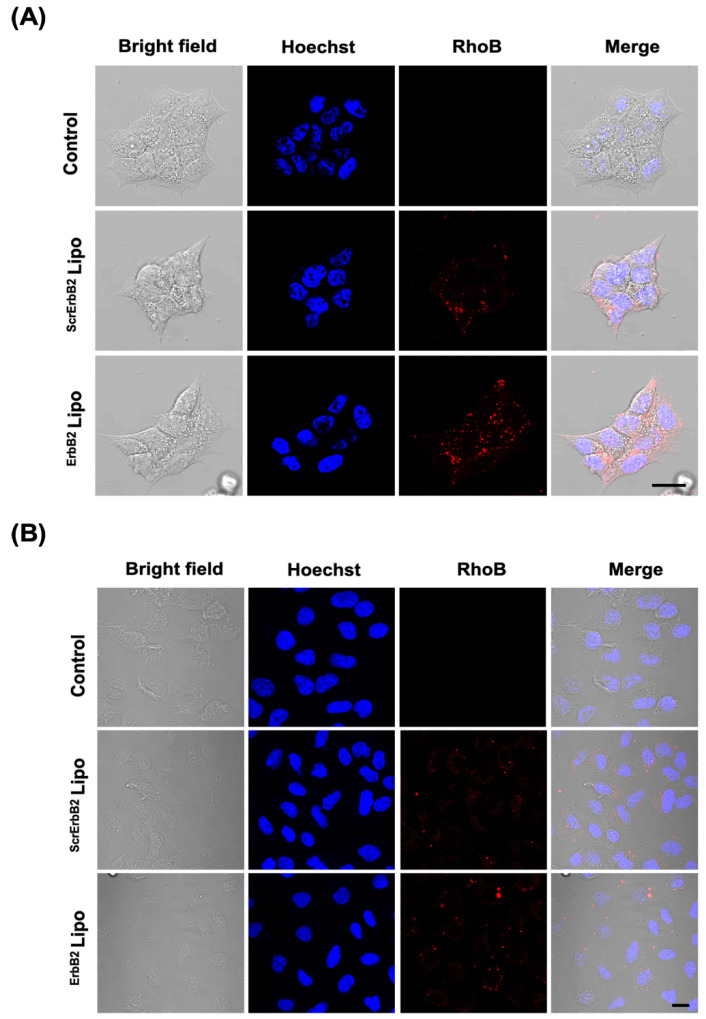
Cellular internalization of the ^ErbB^2Lipo. Representative confocal microscopy images of (**A**) BT-474 and (**B**) MDA-MB-231 cells after incubation for 3 h. Cell nuclei was stained with Hoechst 33342 (blue). Rhodamine B (red) indicated the presence of liposomes. Scale bar: 10 μm.

Using confocal microscopy, BT-474 cells treated with ^ErbB2^Lipo had a stronger rhodamine B fluorescence signal in the cytoplasm when compared with the ^ScrErbB2^Lipo treated-group (Figure 5A). In contrast, in MDA-MB-231 cells, an ErbB2 receptor-negative cell line, which is a weak red fluorescence signal, was observed regardless of the presence of the targeting ligand (Figure 5B). These observations indicated that the ErbB2 peptide synthesized in this study enabled the specific targeting of cancer cells highly expressing ErbB2 on the surface of the cell membrane. Furthermore, ErbB2 receptor target specificity was confirmed using the ErbB2 blocking assay. After 3 h pre-treatment of anti-ErbB2 antibody, cellular internalized ^ErbB2^lipo was reduced when compared to anti-ErbB2 antibody non-treated cells (Figure S4). These results suggest that ^ErbB2^lipo was located in cancer cells through ErbB2 receptor-mediated endocytosis.

Although not mentioned in detail in this study, it would be worthwhile to compare the peptide with the different types of ligands such as an antibody, mini-body, or aptamer for further evaluating and improving the targetability of the peptide. New peptide sequences with better affinity should be discovered in order to surpass trastuzumab, which is binding to the HER2 extracellular domain with an affinity of 0.5 nM [37]. Moreover, according to Chen Q et al., PEG chain length is critical for maximize the genuine function of targeting peptides. The targeting efficiency could be enhanced by adjusting the length of the PEG chain shorter than PEG2000 [38].

### 3.5. Anti-Cancer Activity of ErbB2-Targeting Liposomes

**Figure 6:**
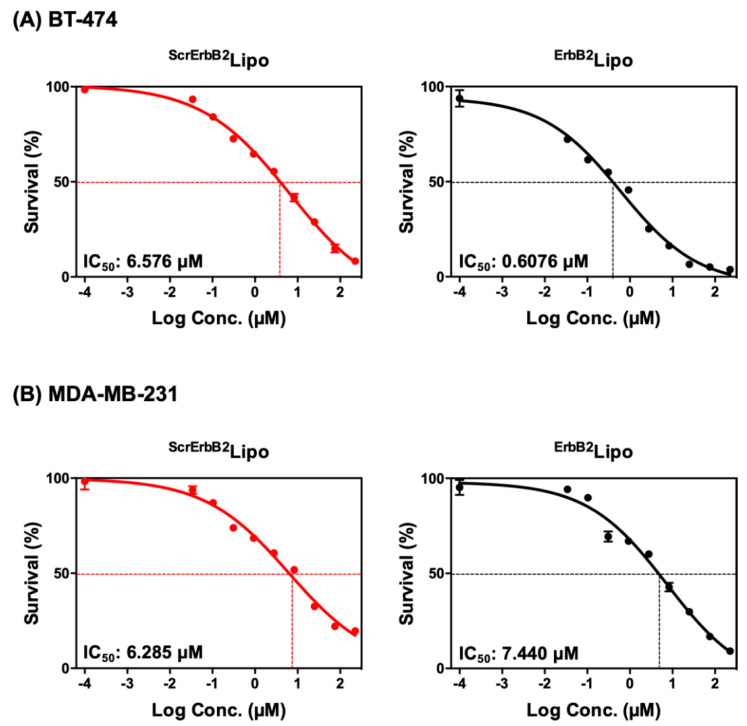
The cytotoxicity of ^ScrErbB2^Lipo and ^ErbB2^Lipo after 72 h in (**A**) BT-474 and (**B**) MDA-MB-231 cells. Results are presented as mean ± SEM (*n* = 8).

To evaluate the anti-cancer activity of rapamycin, BT-474 cells and MDA-MB-231 cells were treated with various concentrations of ^ErbB2^Lipo or ^ScrErbB2^Lipo, and the cell viability was then determined after incubation for 72 h. The MTT assay demonstrated that ^ErbB2^Lipo exerted higher cytotoxicity in the BT-474 cells than ^ScrErbB2^Lipo, which indicated that ^ErbB2^Lipo exhibits good targetability (Figure 6A). The IC_50_ values were 10.8-fold different with values of 6.576 and 0.6076 μM for ^ScrErbB2^Lipo and ^ErbB2^Lipo, respectively. Under the same conditions, no significant differences were observed in MDA-MB-231 cells treated with either ^ErbB2^Lipo or ^ScrErbB2^Lipo (Figure 6B). Some cell death was induced by ^ScrErbB2^Lipo treatment in both BT-474 and MDA-MB-231 cells, and this is because the liposomes can fuse with cell membranes without any targeting ligands [39]. In conclusion, these results indicated that the presence of the targeting peptide enhanced the therapeutic effect of rapamycin in ErbB2-expressing breast cancer cells via targeted drug delivery.

The latest regimens for ErbB2-positive breast cancer patients mostly follow the National Comprehensive Cancer Network clinical practice guidelines, which are widely used as an international standard for cancer treatment. According to the guidelines, trastuzumab and/or pertuzumab treatments are preferred for most ErbB2-positive breast cancer patients. In addition, many clinical trials have shown that the addition of an anthracycline and/or taxane-based chemotherapy results in an improved outcome [40]. Although the use of mTOR inhibitors, such as rapamycin (sirolimus), everolimus, and temsirolimus, are not highly recommended regimens, some patients may benefit from the use of mTOR inhibitors. Drug resistance to trastuzumab is frequently reported, mainly associated with PI3K-AKT3-mTOR signaling activation. This pathway is involved in cell growth and proliferation, and rapamycin treatment can abrogate the signals involved in activation of this pathway. There are several preclinical and clinical trials that have reported promising results using rapamycin in combination with a drug delivery system. The ErbB2-targeting approach is an effective method for drug delivery using an appropriate tumor-specific marker. In the present study, we have developed an efficient rapamycin delivery system, which has the potential to target ErbB2-positive tumors and cause tumor cell death.

## 4. Conclusions

In this study, we designed peptide-conjugated liposomes specifically targeting ErbB2-positive BT-474 breast cancer cells. The ErbB2-targeting peptide and the negative control, ScrErbB2, were synthesized and characterized by HPLC, MALDI-TOF mass spectroscopy, and NMR analysis. The properties of the two peptides were almost identical except for the targeting ability, and the peptide-conjugated liposomes specifically bound to the BT-474 cancer cells. ErbB2-targeting liposomes with rapamycin effectively inhibited the growth of BT-474 cancer cells. In conclusion, liposomes targeting ErbB2 have the potential to be an efficient drug delivery carrier for treating ErbB2-positive breast cancer. These findings provide the basis for ErbB2-targeting liposomes to be further developed for use in both breast cancer imaging and therapy by encapsulating drugs that can inhibit tumor growth in complex biological environments.

## Supplementary Materials

The following are available online at https://www.mdpi.com/1999-4923/12/6/585/s1. Table S1. Fmoc determination. Figure S1. Synthetic chemical structure of DSPE-PEG-ErbB2. Figure S2. Characterization of ^ErbB2^Lipo. (A) Size of ^ErbB2^Lipo under physiological condition (pH 7.4, 6.5 and 5.0, respectively) for 5 days. (B) PDI of ^ErbB2^Lipo under physiological condition (pH 7.4, 6.5 and 5.0, respectively) for 5 days. (C) Encapsulation efficiency (red) and loading efficiency (black) of rapamycin in the ^ErbB2^Lipo. (D) Cumulative release of rapamycin at 37 °C in PBS pH 6.5 and pH 7.4. Figure S3. MDA-MB-231 and BT-474 cellular level of ErbB2 (185 kDa). GAPDH was used as an internal control (37 kDa). Figure S4. B. Representative images of BT-474 incubated with ^ErbB2^lipo under pre-treated or non-treated anti-ErbB2 antibody (5 μg, 3 h). Scale bar, 10 μm.

## Abbreviations

ADC	antibody-drug conjugate
^Bare^Lipo	bare liposomes
CHCA	α-cyano-4-hydroxycinnamic acid
CHEMS	cholesteryl hemisuccinate
DCM	dichloromethane
DLS	dynamic light scattering
DMF	*N*,*N*-dimethylformamide
DSPE-PEG	1,2-distearoyl-sn-glycero-3-phosphoethanolamine-N-[methoxy(polyethylene glycol)-2000
EDC	1-ethyl-3-(3-dimethylaminopropyl) carbodiimide
^ErbB2^Lipo	ErbB2-targeting liposomes
HBTU	3-[bis(dimethylamino)methyliumyl]-3H-benzotriazol-1-oxide hexafluorophosphate
HOBt·H_2_O	1- hydroxybenzotriazole hydrate
HPLC	high-performance liquid chromatography
MALDI-TOF	matrix-assisted laser desorption ionization-time-of-flight
mTOR	mammalian target of rapamycin
NHS	*N*-hydroxysuccinimide
PDC	peptide-drug conjugates
PDI	polydispersity index
PEG	polyethylene glycol
PFA	paraformaldehyde
PPD	piperidine
ScrErbB2	scrambled peptide
^ScrErbB2^Lipo	non-targeting liposomes conjugated with ScrErbB2
SPPS	solid-phase peptide synthesis
TEM	transmission electron microscopy
TIS	triisopropylsilane

## References

[1] DeSantis C.E., Ma J.M., Gaudet M.M., Newman L.A., Miller K.D., Sauer A.G., Jemal A., Siegel R.L. (2019). Breast cancer statistics, 2019. Ca-Cancer J. Clin..

[2] Harari D., Yarden Y. (2000). Molecular mechanisms underlying ErbB2/HER2 action in breast cancer. Oncogene.

[3] Tai W., Mahato R., Cheng K. (2010). The role of HER2 in cancer therapy and targeted drug delivery. J. Control. Release.

[4] Tovey S.M., Brown S., Doughty J.C., Mallon E.A., Cooke T.G., Edwards J. (2009). Poor survival outcomes in HER2-positive breast cancer patients with low-grade, node-negative tumours. Br. J. Cancer.

[5] Masoud V., Pages G. (2017). Targeted therapies in breast cancer: New challenges to fight against resistance. World J. Clin. Oncol..

[6] Chen L., Zhou L.L., Wang C.H., Han Y., Lu Y.L., Liu J., Hu X.C., Yao T.M., Lin Y., Liang S.J. (2019). Tumor-Targeted Drug and CpG Delivery System for Phototherapy and Docetaxel-Enhanced Immunotherapy with Polarization toward M1-Type Macrophages on Triple Negative Breast Cancers. Adv. Mater..

[7] Dadwal A., Baldi A., Narang R.K. (2018). Nanoparticles as carriers for drug delivery in cancer. Artif. Cell Nanomed. Biotechnol..

[8] Mi P., Cabral H., Kataoka K. (2020). Ligand-Installed Nanocarriers toward Precision Therapy. Adv. Mater..

[9] Sun Q.Q., Bi H.T., Wang Z., Li C.X., Wang X.W., Xu J.T., Zhu H., Zhao R.X., He F., Gai S.L. (2019). Hyaluronic acid-targeted and pH-responsive drug delivery system based on metal-organic frameworks for efficient antitumor therapy. Biomaterials.

[10] Yang Y., Zhao W.H., Tan W.W., Lai Z.Q., Fang D., Jiang L., Zuo C.T., Yang N., Lai Y.R. (2019). An Efficient Cell-Targeting Drug Delivery System Based on Aptamer-Modified Mesoporous Silica Nanoparticles. Nanoscale Res. Lett..

[11] Li T., Amari T., Semba K., Yamamoto T., Takeoka S. (2017). Construction and evaluation of pH-sensitive immunoliposomes for enhanced delivery of anticancer drug to ErbB2 over-expressing breast cancer cells. Nanomedicine.

[12] Suleman K., Mushtaq A., Haque E., Badran A., Ajarim D., Tweigeri T., Elashwah A., Shahin A., Khan K., Alsayed A. (2019). Retrospective review of Her2 positive metastatic breast cancer patients who received Pertuzumab and Herceptin as a first line therapy at KFSH&RC (single institute experience) from 2013 to 2016. Breast.

[13] Chen F., Ma K., Madajewski B., Zhuang L., Zhang L., Rickert K., Marelli M., Yoo B., Turker M.Z., Overholtzer M. (2018). Ultrasmall targeted nanoparticles with engineered antibody fragments for imaging detection of HER2-overexpressing breast cancer. Nat. Commun..

[14] Shen Y., Li M., Liu T., Liu J., Xie Y., Zhang J., Xu S., Liu H. (2019). A dual-functional HER2 aptamer-conjugated, pH-activated mesoporous silica nanocarrier-based drug delivery system provides in vitro synergistic cytotoxicity in HER2-positive breast cancer cells. Int. J. Nanomed..

[15] Park J.W., Hong K., Kirpotin D.B., Colbern G., Shalaby R., Baselga J., Shao Y., Nielsen U.B., Marks J.D., Moore D. (2002). Anti-HER2 immunoliposomes: Enhanced efficacy attributable to targeted delivery. Clin. Cancer Res..

[16] Gao Z., Li G., Li X., Zhou J., Duan X., Chen J., Joshi B.P., Kuick R., Khoury B., Thomas D.G. (2017). In vivo near-infrared imaging of ErbB2 expressing breast tumors with dual-axes confocal endomicroscopy using a targeted peptide. Sci. Rep..

[17] Mansoori B., Mohammadi A., Davudian S., Shirjang S., Baradaran B. (2017). The Different Mechanisms of Cancer Drug Resistance: A Brief Review. Adv. Pharm. Bull..

[18] Yellen P., Saqcena M., Salloum D., Feng J., Preda A., Xu L., Rodrik-Outmezguine V., Foster D.A. (2011). High-dose rapamycin induces apoptosis in human cancer cells by dissociating mTOR complex 1 and suppressing phosphorylation of 4E-BP1. Cell Cycle.

[19] Wang L.H., Chan J.L., Li W. (2007). Rapamycin together with herceptin significantly increased anti-tumor efficacy compared to either alone in ErbB2 over expressing breast cancer cells. Int. J. Cancer.

[20] Decker T., Marschner N., Muendlein A., Welt A., Hagen V., Rauh J., Schroder H., Jaehnig P., Potthoff K., Lerchenmuller C. (2019). VicTORia: A randomised phase II study to compare vinorelbine in combination with the mTOR inhibitor everolimus versus vinorelbine monotherapy for second-line chemotherapy in advanced HER2-negative breast cancer. Breast Cancer Res. Treat..

[21] Pascual J., Turner N.C. (2019). Targeting the PI3-kinase pathway in triple-negative breast cancer. Ann. Oncol..

[22] Schmid P., Zaiss M., Harper-Wynne C., Ferreira M., Dubey S., Chan S., Makris A., Nemsadze G., Brunt A.M., Kuemmel S. (2019). Fulvestrant Plus Vistusertib vs Fulvestrant Plus Everolimus vs Fulvestrant Alone for Women With Hormone Receptor-Positive Metastatic Breast Cancer: The MANTA Phase 2 Randomized Clinical Trial. JAMA Oncol..

[23] Kim M.W., Jeong H.Y., Kang S.J., Jeong I.H., Choi M.J., You Y.M., Im C.S., Song I.H., Lee T.S., Lee J.S. (2019). Anti-EGF Receptor Aptamer-Guided Co-Delivery of Anti-Cancer siRNAs and Quantum Dots for Theranostics of Triple-Negative Breast Cancer. Theranostics.

[24] Sachdeva S., Joo H., Tsai J., Jasti B., Li X. (2019). A Rational Approach for Creating Peptides Mimicking Antibody Binding. Sci. Rep..

[25] Amblard M., Fehrentz J.A., Martinez J., Subra G. (2006). Methods and protocols of modern solid phase Peptide synthesis. Mol. Biotechnol..

[26] Zhang Y., Mintzer E., Uhrich K.E. (2016). Synthesis and characterization of PEGylated bolaamphiphiles with enhanced retention in liposomes. J. Colloid Interface Sci..

[27] Fang Y., Xue J., Gao S., Lu A., Yang D., Jiang H., He Y., Shi K. (2017). Cleavable PEGylation: A strategy for overcoming the “PEG dilemma” in efficient drug delivery. Drug Deliv..

[28] Ahmad Z., Shah A., Siddiq M., Kraatz H.B. (2014). Polymeric micelles as drug delivery vehicles. Rsc Adv..

[29] Eloy J.O., Petrilli R., Topan J.F., Antonio H.M.R., Barcellos J.P.A., Chesca D.L., Serafini L.N., Tiezzi D.G., Lee R.J., Marchetti J.M. (2016). Co-loaded paclitaxel/rapamycin liposomes: Development, characterization and in vitro and in vivo evaluation for breast cancer therapy. Colloid Surface B.

[30] Lucas A.T., Price L.S.L., Schorzman A.N., Storrie M., Piscitelli J.A., Razo J., Zamboni W.C. (2018). Factors Affecting the Pharmacology of Antibody-Drug Conjugates. Antibodies (Basel).

[31] Vrettos E.I., Mezo G., Tzakos A.G. (2018). On the design principles of peptide-drug conjugates for targeted drug delivery to the malignant tumor site. Beilstein J. Org. Chem..

[32] Ferri N., Bellosta S., Baldessin L., Boccia D., Racagni G., Corsini A. (2016). Pharmacokinetics interactions of monoclonal antibodies. Pharmacol. Res..

[33] Javier D.J., Nitin N., Levy M., Ellington A., Richards-Kortum R. (2008). Aptamer-targeted gold nanoparticles as molecular-specific contrast agents for reflectance imaging. Bioconjug. Chem..

[34] Danhier F. (2016). To exploit the tumor microenvironment: Since the EPR effect fails in the clinic, what is the future of nanomedicine?. J. Control. Release.

[35] Xie J., Wang X., Proud C.G. (2016). mTOR inhibitors in cancer therapy. F1000Research.

[36] Kenny P.A., Lee G.Y., Myers C.A., Neve R.M., Semeiks J.R., Spellman P.T., Lorenz K., Lee E.H., Barcellos-Hoff M.H., Petersen O.W. (2007). The morphologies of breast cancer cell lines in three-dimensional assays correlate with their profiles of gene expression. Mol. Oncol..

[37] Kute T., Lack C.M., Willingham M., Bishwokama B., Williams H., Barrett K., Mitchell T., Vaughn J.P. (2004). Development of Herceptin resistance in breast cancer cells. Cytom. A.

[38] Chen Q., Yu S., Zhang D., Zhang W., Zhang H., Zou J., Mao Z., Yuan Y., Gao C., Liu R. (2019). Impact of Antifouling PEG Layer on the Performance of Functional Peptides in Regulating Cell Behaviors. J. Am. Chem. Soc..

[39] Foldvari M. (2015). Observations of membrane fusion in a liposome dispersion: The missing fusion intermediate?. F1000Research.

[40] Telli M.L., Gradishar W.J., Ward J.H. (2019). NCCN Guidelines Updates: Breast Cancer. J. Natl. Compr. Cancer Netw..

