# Supplementary material for: Development of ErbB2-Targeting Liposomes for Enhancing Drug Delivery to ErbB2-Positive Breast Cancer
Source: Pharmaceutics. 2020 Jun 24;12(6):585. doi: 10.3390/pharmaceutics12060585 (PMC7356551; doi:10.3390/pharmaceutics12060585)
Supplement: Supplementary file 1 [file pharmaceutics-12-00585-s001.pdf]

pharmaceutics

Supplementary Materials: Development of ErbB2-Targeting Liposomes for Enhancing Drug Delivery to ErbB2-Positive Breast Cancer

Sho Ueno, Min Woo Kim, Gibok Lee, Yong Il Park, Takuro Niidome and Ruda Lee

Table S1. Fmoc determination.

> ErbB2 peptide

Amino acid	Volume[mL]	Abs[-]	mmol	Introduction rate[%]
Arg(Pbf)	14.0	0.799	0.1883	94.15
Lys(boc)	14.0	0.843	0.2080	104.00
Phe	14.0	0.662	0.2049	102.45
Asn(Trt)	14.0	0.558	0.2221	111.05
Ser(tBu)	14.0	0.666	0.2112	105.60
Pro	14.0	0.589	0.2321	116.05
Pro	14.0	0.613	0.2197	109.85

> ScrErbB2 peptide

Amino acid	Volume[mL]	Abs[-]	mmol	Introduction rate[%]
Phe	14.0	1.330	0.2116	105.79
Arg(Pbf)	14.0	1.290	0.2098	104.91
Pro	14.0	1.277	0.2016	100.81
Asn(Trt)	14.0	1.737	0.2098	104.89
Pro	14.0	1.691	0.2307	115.37
Ser(tBu)	14.0	1.439	0.2269	113.46
Lys(boc)	14.0	1.457	0.2028	101.42

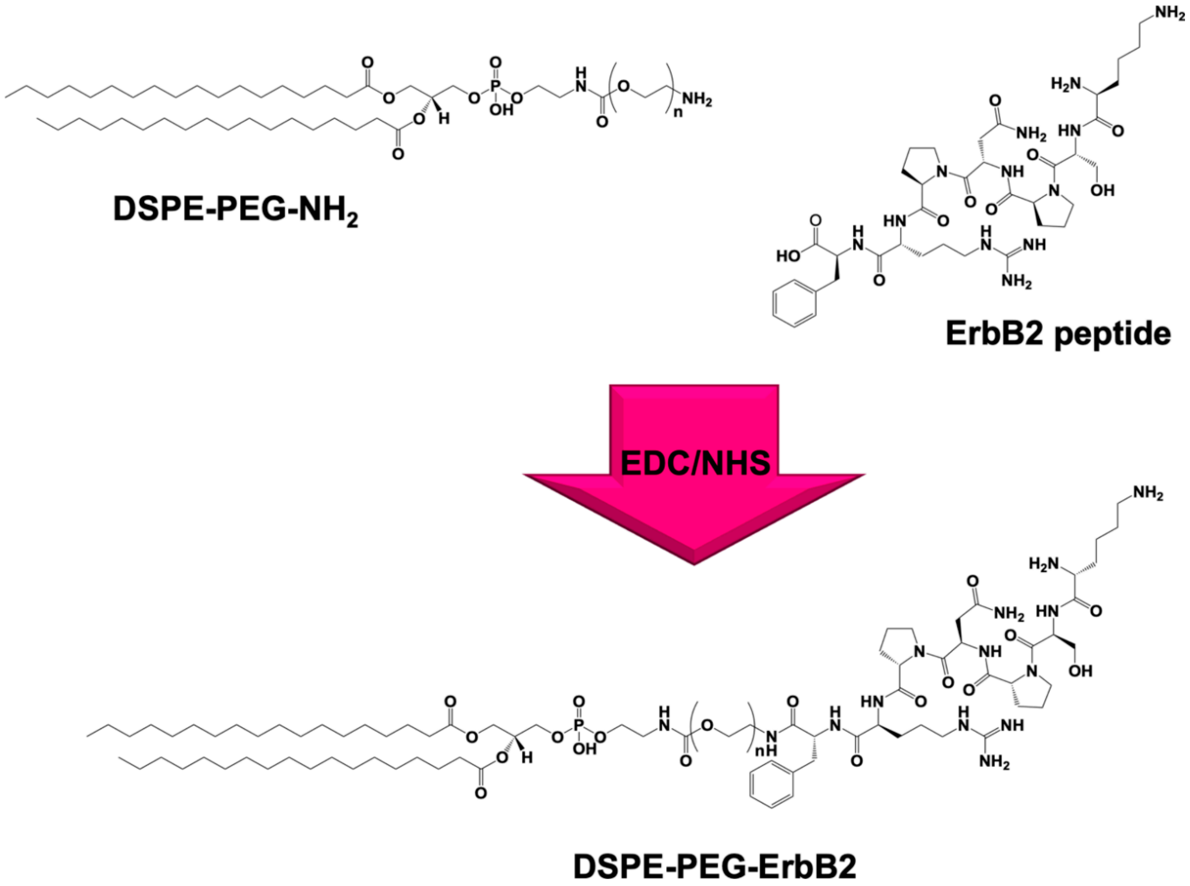

Figure S1. Synthetic chemical structure of DSPE-PEG-ErbB2.

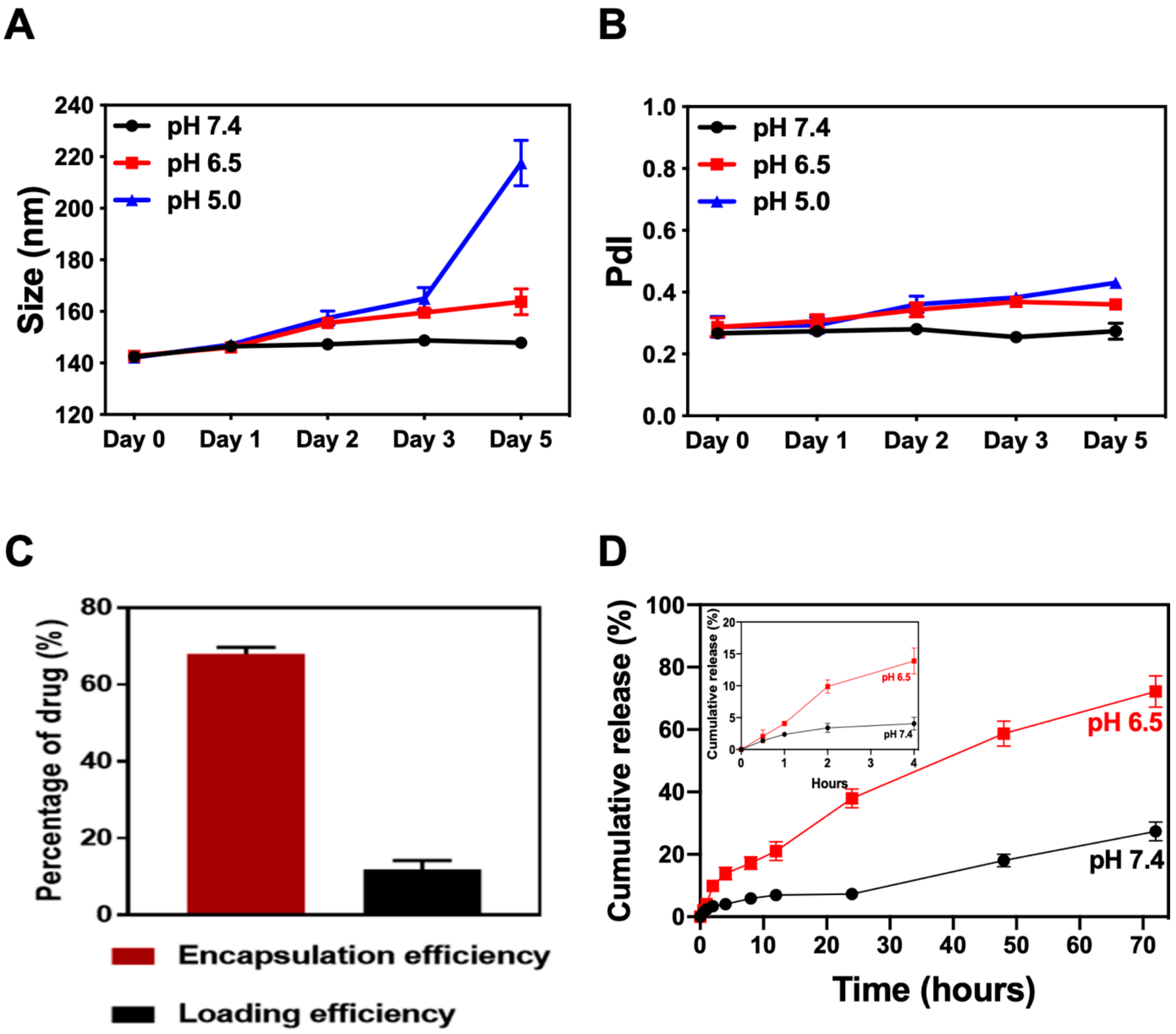

Figure 2. Characterization of ErbB2Lipo. (**A**) Size of ErbB2Lipo under PBS (pH 7.4, 6.5 and 5.0, respectively) for 5 days. (**B**) PdI of ErbB2Lipo under PBS (pH 7.4, 6.5 and 5.0, respectively) for 5 days. (**C**) Encapsulation efficiency (red) and loading efficiency (black) of Rapa in the ErbB2Lipo. (**D**) Cumulative release of Rapa at 37 °C in PBS pH 6.5 and pH 7.4.

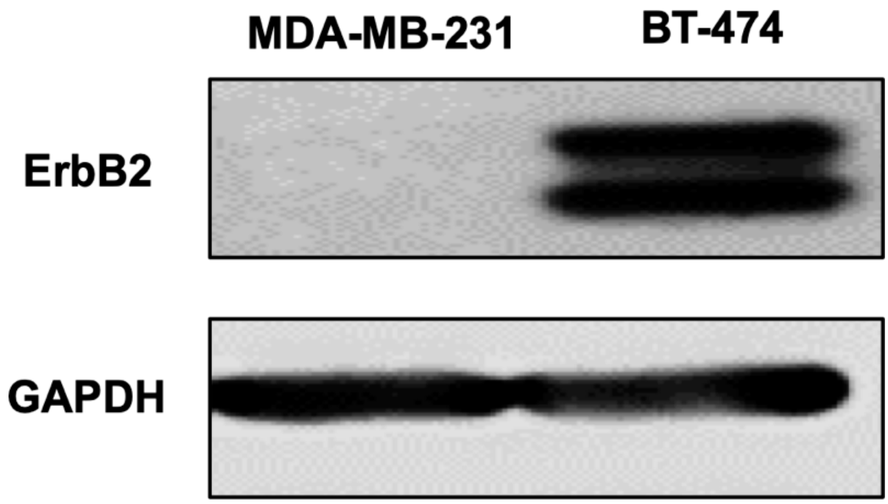

Figure S3. MDA-MB-231 and BT-474 cellular level of ErbB2 (185 kDa). GAPDH was used as an internal control (37 kDa). Scale bar

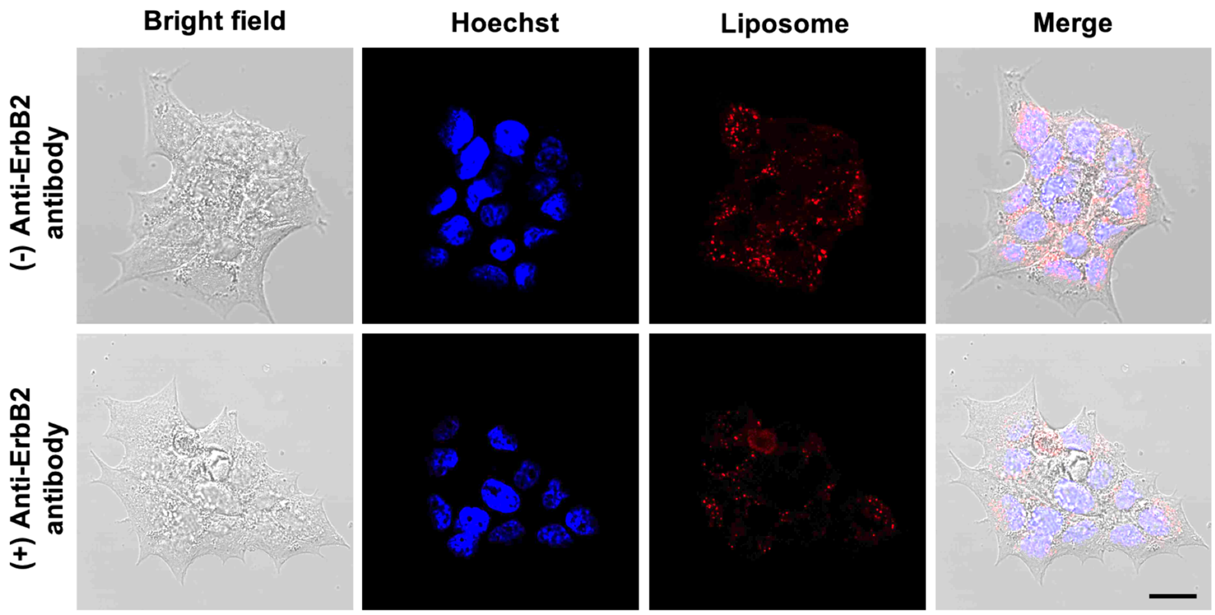

Figure S4. Representative images of BT-474 incubated with ^{ErbB2}lipo under pre-treated or non-treated anti-ErbB2 antibody (5 μg, 3 h). Scale bar, 10 μm.
